# Psychological readiness and adaptation to performance stress in female athletes: the mediating role of sport confidence between athletic identity and mental toughness

**DOI:** 10.1186/s13102-026-01621-z

**Published:** 2026-03-04

**Authors:** Hatice Gezer, Murat Korucuk

**Affiliations:** Faculty of Sports Sciences, Kafkas University, Kars, Turkey

**Keywords:** Psychological readiness, Athletic identity, Sport confidence, Mental toughness, Female athletes, Structural equation modeling

## Abstract

**Background:**

Psychological readiness is increasingly recognized as a critical component of training adaptation, performance sustainability, and recovery processes in female athletes. Beyond physical capacity, internal psychological resources such as athletic identity, sport confidence, and mental toughness play a key role in athletes’ ability to tolerate performance-related stress, maintain emotional control, and adapt to demanding training and competition environments. However, the interrelationships among these constructs and their contribution to psychological readiness remain insufficiently examined in female athletic populations. In this study, psychological readiness is conceptualized as an adaptive psychological capacity reflected through athletic identity, sport confidence, and mental toughness.

**Methods:**

This cross-sectional correlational study included 449 female university athletes participating in individual and team sports. Data were collected using the Athletic Identity Measurement Scale (AIMS), Sport Mental Toughness Questionnaire (SMTQ), and Sport Confidence Inventory (SCI). Construct validity and reliability were confirmed through confirmatory factor analysis (CFI = 0.95–0.99; RMSEA = 0.025–0.077; Cronbach’s α = 0.89–0.94). Relationships among variables were examined using Pearson correlation analysis and structural equation modeling to test direct and mediating effects.

**Results:**

Athletes demonstrated high levels of athletic identity (M = 3.69), sport confidence (M = 3.73), and mental toughness (M = 3.57). All constructs were positively and strongly correlated (*r* = .67–0.70, *p* < .001). Structural equation modeling revealed that athletic identity significantly predicted both sport confidence and mental toughness (*p* < .001). Sport confidence also exerted a significant positive effect on mental toughness (*p* < .001). When included as a mediator, sport confidence partially reduced—but did not eliminate—the direct effect of athletic identity on mental toughness, indicating a pattern consistent with partial statistical mediation. Model fit indices indicated satisfactory model fit (CFI = 0.94–0.96; RMSEA = 0.05–0.06).

**Conclusions:**

Athletic identity contributes to mental toughness both directly and indirectly through sport confidence in female athletes. These findings suggest that identity- and confidence-based psychological resources play an important role in psychological readiness and adaptation to performance-related stress. From an applied sport science and rehabilitation perspective, monitoring athletic identity and sport confidence may support training adaptation, psychological readiness, and informed return-to-play decision-making in female athletes. Given the cross-sectional design, these findings should be interpreted as associative rather than causal, and practical implications should be applied with appropriate caution.

## Introduction

Women’s participation in competitive sport has increased significantly in recent decades, making it especially important to understand the psychological factors that shape their performance, resilience, and recovery [18].

Contemporary sport psychology consistently emphasizes that psychological characteristics—particularly identity, confidence, and mental toughness—play decisive roles in how athletes manage stress, sustain motivation, and maintain stable performance [11, 14]. Despite this growing recognition, sport science and sports medicine literature continue to underrepresent the psychological experiences of female athletes [26]. This underrepresentation is particularly important because female athletes are more likely to encounter gender-specific sociocultural expectations, identity-related evaluation pressures, and role conflict dynamics that may shape psychological readiness and resilience differently from male athlete populations. Female athletes often operate under sociocultural and familial expectations that shape how they perceive themselves, regulate emotions, and interpret competitive demands [18]. These pressures influence motivation, stress tolerance, and psychological readiness, indicating the need for gender-sensitive conceptual models that consider these contextual influences [14]. Athletic identity (AI) is one such foundational construct. AI denotes the extent to which individuals assimilate the athlete role and obtain competence, purpose, and self-esteem from participation in sports [3]. Stronger identity structures are associated with intrinsic motivation, persistence, and greater behavioral consistency during stress [25, 29]. Research further shows that AI develops through perceived competence, social reinforcement, and environmental expectations within sport settings [35]. For female athletes, identity formation frequently intersects with societal norms concerning femininity, appearance, and role expectations, rendering AI an essential psychological anchor for sustaining emotional stability and performance-oriented behavior [18, 26]. Mental toughness (MT) constitutes another essential component of performance. MT encompasses the ability to regulate emotions, remain focused under pressure, manage adversity, and maintain persistence in challenging situations. [20, 28, 32].

Mental toughness has gained increasing attention in sports science and sports medicine as a psychological factor influencing athletes’ tolerance to training load, adaptation to physical stress, and consistency under demanding performance conditions. Beyond competitive outcomes, higher levels of mental toughness have been associated with improved emotional regulation, reduced stress reactivity, and greater persistence during periods of intensified training or performance fluctuation. From an applied perspective, these characteristics are particularly relevant for maintaining training continuity, minimizing maladaptive stress responses, and supporting athletes’ capacity to adapt to cumulative physical and psychological demands. Accordingly, mental toughness is now considered an important component of psychological readiness within applied sport science frameworks. This perspective has shifted mental toughness from a purely performance-related construct to a broader indicator of athletes’ capacity to adapt to sustained physical and psychological stress.

In recent years, psychological readiness has also been recognized as a central factor in rehabilitation and return-to-play processes. While physical recovery remains essential, insufficient psychological readiness—characterized by reduced confidence, emotional instability, or disrupted athletic identity—may compromise decision-making, adherence to rehabilitation protocols, and safe return to sport. Female athletes may experience distinct psychological challenges related to evaluative pressures and identity-related stressors. Within this context, psychological constructs such as athletic identity and sport confidence may function as internal regulatory resources that support mental toughness and psychological readiness during rehabilitation and return-to-play processes. Understanding the interaction of these psychological mechanisms may provide valuable insights for applied sport science and rehabilitation practice. Examining these relationships may also inform multidisciplinary approaches in sports medicine, rehabilitation, and performance support settings. Studies involving university and elite female athletes indicate that those with stronger MT display greater emotional regulation, psychological consistency, and performance stability [6, 24]. Additionally, engagement in structured physical activity has been shown to improve mental toughness and stress management across genders, with notable effects among women [1].

Sport confidence (SC) functions as a key mechanism that translates deeper psychological traits, such as identity, into observable performance behaviors. SC reflects athletes’ beliefs in their ability to succeed and is closely linked to perceived competence, cognitive efficiency, and emotional control during competitive tasks [36]. Confident athletes typically demonstrate stronger decision-making, more effective attentional control, and better coping under pressure—features that correspond closely with the foundations of mental toughness [6, 11]. Although AI, SC, and MT have each been examined extensively as individual constructs, research integrating these variables within a single theoretical framework remains limited for female athletes. Little empirical evidence clarifies whether sport confidence mediates the relationship between athletic identity and mental toughness—a meaningful gap considering the unique psychological and sociocultural pressures women face in sport [14, 18].

**Fig. 1:**
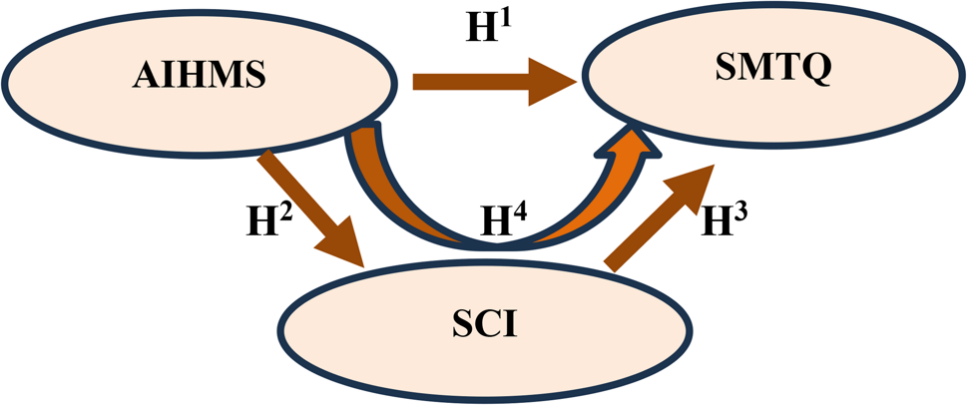
Research model

To address this gap, the present study investigates the direct and indirect pathways connecting athletic identity, sport confidence, and mental toughness in a large national sample of female athletes. Specifically, it tests whether athletic identity predicts sport confidence and mental toughness (H1–H2), whether sport confidence predicts mental toughness (H3), and whether sport confidence mediates the relationship between athletic identity and mental toughness (H4). By clarifying these pathways, the study proposes a gender-sensitive psychological model that advances understanding of resilience, performance readiness, and psychological functioning in female athletes. Based on the theoretical framework and existing literature, a conceptual research model was developed to examine the relationships among athletic identity, sport confidence, and mental toughness (Fig. 1).

### H1. Athletic identity → mental toughness

#### H1: The athletic identity of female athletes participating in individual and team sports affects their mental toughness

Athletic identity reflects the degree to which individuals internalize the athlete role and derive competence and self-worth from sport participation. Strong identity structures are typically associated with intrinsic motivation, behavioral consistency, and emotional regulation—characteristics that align with the core components of mental toughness. Because female athletes often encounter additional sociocultural pressures related to appearance, legitimacy, and performance expectations, identity may play a particularly central role in shaping their resilience. Accordingly, higher athletic identity is expected to predict stronger mental toughness.

### H2. Athletic identity → sport confidence

#### H2: The athletic identity of female athletes participating in individual and team sports affects their level of sport confidence

Athletic identity functions as a psychological foundation that shapes beliefs about competence and capability. When athletes strongly internalize their role, they tend to adopt more positive evaluations of their abilities, maintain higher self-worth, and engage more confidently in performance contexts. These processes parallel the structure of sport confidence, which represents athletes’ expectations for successful performance. Therefore, athletic identity is expected to predict sport confidence positively.

### H3. Sport confidence → mental toughness

#### H3: The sport confidence levels of female athletes participating in individual and team sports affect their mental toughness

Sport confidence is important because it influences how athletes interpret and respond to competitive demands. Confident athletes typically demonstrate greater emotional control, stronger attentional focus, and more persistent effort—behaviors that mirror the essential components of mental toughness.

Given that female athletes may experience heightened social and evaluative pressures; confidence becomes especially important for sustaining resilience under stress. Thus, higher sport confidence is expected to enhance mental toughness.

### H4. Mediating role of sport confidence

#### H4: Sport confidence mediates the effect of athletic identity on the mental toughness of female athletes participating in individual and team sports

Theoretical models suggest that identity-based self-perceptions influence competence beliefs, which subsequently shape performance-related resilience. Within this framework, athletic identity may strengthen mental toughness indirectly by first enhancing sport confidence.

As athletes gain a better understanding of their identity in sports, they strengthen their beliefs about their skills and readiness. These confidence-related processes then facilitate emotional stability, attentional control, and persistence—all key components of mental toughness. Therefore, sport confidence is expected to serve as a mediating mechanism between athletic identity and mental toughness.

## Method

### Research design

This study employed a cross-sectional correlational design to examine the relationships among athletic identity, mental toughness, and sport confidence in female athletes participating in individual and team sports. Correlational designs allow researchers to assess the strength and direction of associations among naturally occurring variables without experimental manipulation [4, 23, 33, 34].

### Population and sample

Data were collected from female athletes participating in individual and team sports during national-level sporting events and competitions held across multiple provinces in Turkey in 2025. Based on a 5% margin of error and a 95% confidence level, the minimum required sample size was calculated as 384 using standard sampling formulas recommended in methodological literature [8, 22]. To enhance statistical power and representativeness, data were collected from a larger sample using face-to-face administration. Initially, 511 questionnaires were obtained; however, 62 questionnaires were excluded due to incomplete responses or inconsistencies in the data. The final analyses were conducted with a total of 449 female athletes.

Due to the field-based nature of data collection during competitive events, a probability-based sampling procedure was not feasible. Therefore, a convenience sampling approach based on participant availability was employed. To improve sample diversity, athletes were recruited across multiple provinces, competition settings, and sport branches.

Because a convenience sampling approach was used, the generalizability of the findings should be interpreted with caution, particularly across different competitive levels and cultural contexts.

Gender served as the primary inclusion criterion, and only female athletes were included. This approach reflects the limited availability of gender-specific research in the literature and the need to better understand the relationships among athletic identity, sport confidence, and mental toughness in women. Restricting the sample to female athletes also enhanced internal validity by reducing variance attributable to gender-related differences.

Of the participants, 56.6% (*n* = 254) competed in individual sports and 43.4% (*n* = 195) in team sports. Regarding sport experience, 55.7% (*n* = 250) had participated for ≤ 5 years, 33.4% (*n* = 150) for 6–10 years, 8.0% (*n* = 36) for 11–15 years, and 2.9% (*n* = 16) for more than 15 years.

### Data collection tools

#### Athletic Identity Measurement Scale (AIMS)

The Athletic Identity Measurement Scale (AIMS) comprises 7 items across three subscales: Social Identity (3 items), Exclusivity (2 items), and Negative Affectivity (2 items).

The scale was originally developed and validated in English and subsequently adapted into Turkish [3, 27]. Reliability analyses of the Turkish version reported an internal consistency coefficient of α = 0.81.

#### Sport Mental Toughness Questionnaire (SMTQ)

The Sport Mental Toughness Questionnaire (SMTQ) includes 14 items distributed across three subscales: Confidence (6 items), Constancy (4 items), and Control (4 items). The instrument was originally developed in English and later adapted into Turkish. The Turkish adaptation demonstrated acceptable internal consistency, with a reported Cronbach’s alpha coefficient of α = 0.84 [2, 28].

#### Sport Confidence Inventory (SCI)

The Sport Confidence Inventory (SCI) consists of 13 items and is structured as a unidimensional scale. The instrument was originally developed in English and subsequently adapted into Turkish. The original version demonstrated high internal consistency, with a reported Cronbach’s alpha coefficient of α = 0.93 [7, 36].

All measurement instruments were administered using a five-point Likert-type scale ranging from 1 (strongly disagree) to 5 (strongly agree). Mean score interpretation intervals were defined as follows: 1.00–1.80 = very low, 1.81–2.60 = low, 2.61–3.40 = moderate, 3.41–4.20 = high, and 4.21–5.00 = very high.

### Validity and reliability procedures

Construct validity and reliability of the measurement instruments were evaluated prior to hypothesis testing. Confirmatory factor analyses (CFA) were conducted for all scales, and model fit indices along with internal consistency coefficients were calculated.

**Fig. 2:**
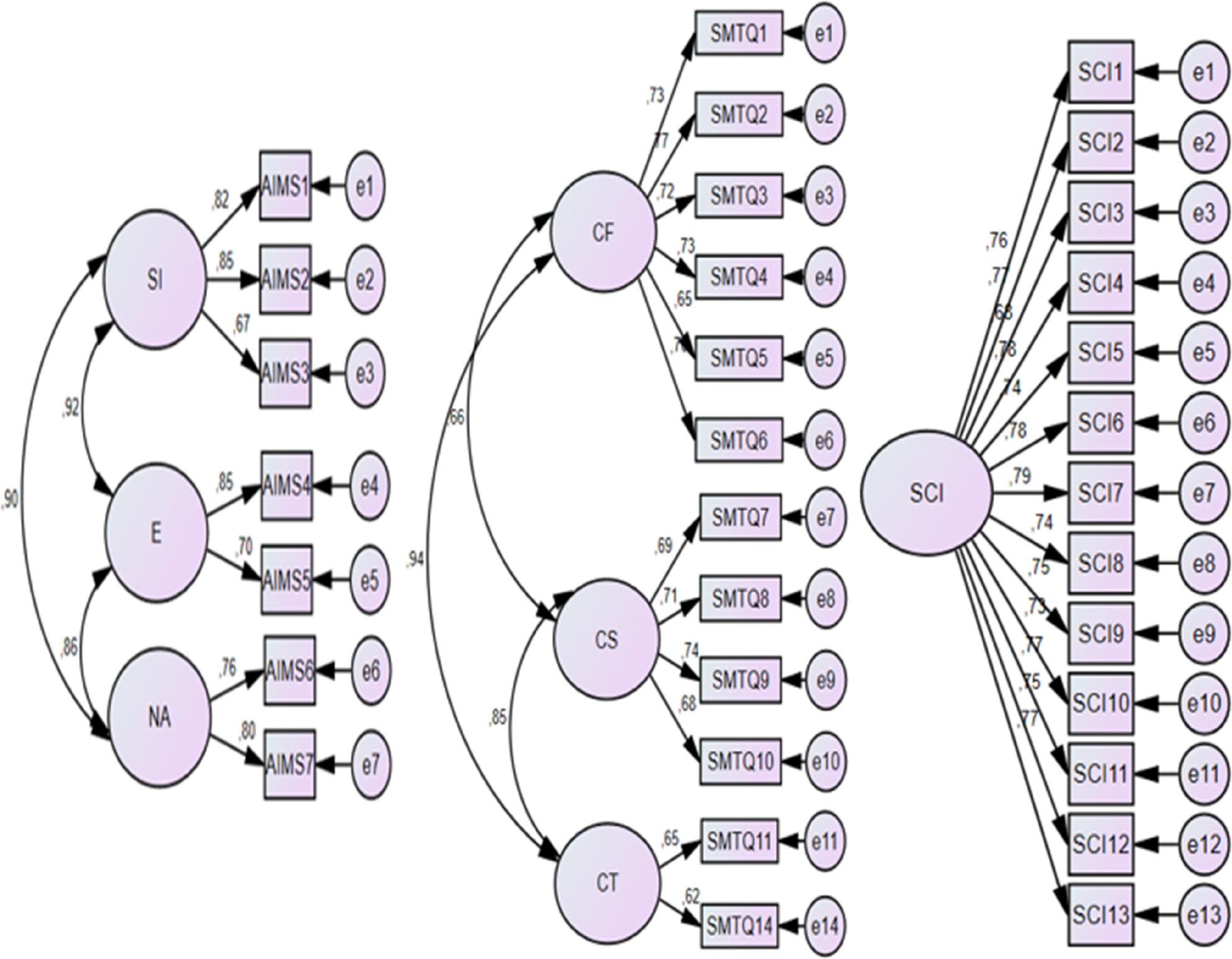
Confirmatory factor analysis models for AIMS, SMTQ, and SCI

CFA models are presented in Fig. 2, and detailed fit indices and reliability statistics are reported in Table 1.

**Table 1:** Fit indices and reliability values for AIMS, SMTQ, and SCI

Fit Indices	Reference Range		Results			Evaluation		
	Good	Acceptable	AIMS	SMTQ	SCI	AIMS	SMTQ	SCI
CMIN/DF	0 < χ²/df ≤ 3	3 < χ²/df ≤ 5	1.277	2.625	3.662	Good	Good	Acceptable
RMSEA	0 ≤ RMSEA≤ 0.05	0.05 < RMSEA≤ 0.08	0.025	0.060	0.077	Good	Acceptable	Acceptable
GFI	0.90 < GFI ≤ 1.00	0.80 < GFI≤ 0.90	0.991	0.950	0.921	Good	Good	Good
AGFI	0.90 < AGFI ≤ 1.00	0.80 < AGFI≤ 0.90	0.977	0.924	0.890	Good	Acceptable	Acceptable
CFI	0.95 < CFI ≤ 1.00	0.90 < CFI≤ 0.94	0.998	0.962	0.954	Good	Good	Good
RMR	0 ≤ RMR≤ 0.05	0.05 < RMR≤ 0.10	0.016	0.035	0.031	Good	Good	Good
TLI	0.95 < TLI ≤ 1.00	0.90 < TLI≤ 0.94	0.996	0.951	0.945	Good	Good	Good
NFI	0.95 < NFI ≤ 1.00	0.90 < NFI≤ 0.94	0.991	0.941	0.938	Good	Good	Good
df	—	—	11	51	65	—	—	—
CMIN	—	—	14.043	133.865	238.029	—	—	—
Cronbach Alpha	0.60 ≤ α ≥ 0.80 Acceptable 0.80 ≤ α ≥ 1.00 Highly Reliable		0.898	0.894	0.945	High	High	High

*Abbreviations*: *AIMS* Athletic Identity Measurement Scale, *SMTQ* Sport Mental Toughness Questionnaire, *SCI* Sport Confidence Inventory, *CFI* Comparative Fit Index, *TLI* Tucker–Lewis Index, *RMSEA* Root Mean Square Error of Approximation, *χ²/df* chi-square divided by degrees of freedom, *α* Cronbach’s alpha. The determination of reference ranges is based on previous literature [5, 12, 15–18, 29, 34]

Based on the CFA results, the AIMS and SCI were retained in their original forms. For the SMTQ, items 12 and 13, both belonging to the Control subscale, were removed due to low factor loadings (< 0.50). The revised 12-item SMTQ demonstrated improved model fit indices, which were deemed acceptable for subsequent analyses (Table 1). Although this item-level modification improved statistical model fit, it may slightly limit strict comparability with the original SMTQ structure, and findings should be interpreted with this consideration in mind.

Overall, the CFA results, model fit indices, and reliability coefficients indicated that the measurement models exhibited satisfactory construct validity and internal consistency for use in the present study.

### Data analysis

Prior to hypothesis testing, preliminary analyses were conducted to ensure the suitability of the data for subsequent statistical procedures. Confirmatory factor analyses and internal consistency coefficients were first examined to evaluate the measurement properties of the instruments (Table 1; Fig. 2).

Data distribution was then assessed using descriptive statistics (mean and standard deviation), skewness and kurtosis values, visual inspection of distribution plots, and normality tests (Kolmogorov–Smirnov and Shapiro–Wilk). The results of these preliminary analyses are presented in Table 2.

**Table 2:** Preliminary data checks

SCALES	Kolmogorov Smirnov			Shapiro Wilk			Skewness	Kurtosis	$\:\stackrel{-}{\boldsymbol{X}}$	SD
	Stat	df	*p*	Stat	df	*p*				
AIMS	0.086	448	0.164	0.968	448	0.13	− 0.467	0.060	3.70	0.82
SMTQ	0.075	448	0.098	0.973	448	0.09	− 0.078	0.230	3.57	0.62
SCI	0.067	448	0.072	0.974	448	0.06	− 0.146	− 0.091	3.73	0.73

*Abbreviations*: *AIMS* Athletic Identity Measurement Scale, *SMTQ* Sport Mental Toughness Questionnaire, *SCI* Sport Confidence Inventory, *CFI* Comparative Fit Index, *TLI* Tucker–Lewis Index, *RMSEA* Root Mean Square Error of Approximation, *χ²/df* chi-square divided by degrees of freedom, *α* Cronbach’s alpha

Skewness and kurtosis values for all variables fell within the acceptable range of − 1 to + 1, indicating approximate normal distribution, consistent with commonly accepted methodological criteria [9, 31].

Based on these findings, parametric statistical techniques were deemed appropriate for subsequent analyses [31]. Pearson correlation coefficients (r) were calculated to examine the relationships among athletic identity, mental toughness, and sport confidence. Correlation analysis was conducted as a preliminary step to evaluate bivariate association patterns and ensure that the relationships among variables were suitable for subsequent structural model testing. Structural equation modeling (SEM) was then employed to test the hypothesized direct and indirect relationships among the study variables [19]. Model fit was evaluated using established goodness-of-fit indices, with reference values presented in Table 1. All statistical analyses were conducted using SPSS version 22 and AMOS software. Ethical principles for scientific research were observed throughout the study.

## Results

Table 3 presents the mean (M) and standard deviation (SD) values for the sub-dimensions and totals of the data collection tools (AIMS, SMTQ, and SCI) to determine the athletic identity, mental toughness, and sport confidence levels of female athletes participating in individual and team sports.

**Table 3:** AIMS, SMTQ, and SCI levels

SCALES	Subscales	*n*	$\:\stackrel{-}{\boldsymbol{X}}$	SD	Interpretation
AIMS	Social Identity-SI (3 Item)	449	3.65	0.92	High
	Exclusivity-E (2 Item)		3.61	0.90	High
	Negative Affectivity-NA (2 Item)		3.85	0.94	High
	Total		3.69	0.82	High
SMTQ	Confidence-CF (6 Item)	449	3.64	0.73	High
	Constancy-CS (4 Item)		3.43	0.73	High
	Control-CT (4 Item)		3.59	0.79	High
	Total		3.57	0.62	High
SCI	Total	449	3.73	0.73	High

*Abbreviations*: *AIMS* Athletic Identity Measurement Scale, *SMTQ* Sport Mental Toughness Questionnaire, *SCI* Sport Confidence Inventory, *CFI* Comparative Fit Index, *TLI* Tucker–Lewis Index, *RMSEA* Root Mean Square Error of Approximation, *χ²/df* Chi-square divided by degrees of freedom, *α* Cronbach’s alpha

Table 3 presents the descriptive statistics for the AIMS, SMTQ, and SCI and their respective subscales. Mean scores ranged from 3.43 to 3.85, while standard deviation values ranged between 0.62 and 0.94. According to the predefined score interpretation criteria, mean values for all scales and subscales were within the high score range. The findings of the Pearson Correlation analysis conducted to determine the relationship between the athletic identity, mental toughness, and sport confidence levels of female athletes participating in individual and team sports are presented in Table 4.

**Table 4:** Levels of relationship between AIMS, SMTQ, and SCI

SCALES		AIMS	SMTQ	SCI
AIMS	*r*	1		
	*p*	-		
SMTQ	*r*	0.704^**^	1	
	*p*	0.00	-	
SCI	*r*	0.683^**^	0.674^**^	1
	*p*	0.00	0.00	-

*Abbreviations*: *AIMS* Athletic Identity Measurement Scale, *SMTQ* Sport Mental Toughness Questionnaire, *SCI* Sport Confidence Inventory, *CFI* Comparative Fit Index, *TLI* Tucker–Lewis Index, *RMSEA* Root Mean Square Error of Approximation, *χ²/df *Chi-square divided by degrees of freedom, *α* Cronbach’s alpha

** p < .01

Pearson correlation analyses examining the relationships among athletic identity, mental toughness, and sport confidence are presented in Table 4. Athletic identity was positively and strongly correlated with mental toughness (*r* = .704, *p* < .01) and positively correlated with sport confidence (*r* = .683, *p* < .01). In addition, sport confidence showed a positive and moderate correlation with mental toughness (*r* = .674, *p* < .01). Overall, the results indicate statistically significant positive associations among the study variables. Although the correlations and standardized path coefficients are relatively high, confirmatory factor analyses supported the distinct factor structures of the measurement models, indicating acceptable discriminant validity at the latent construct level.

### Findings related to H1

**Fig. 3:**
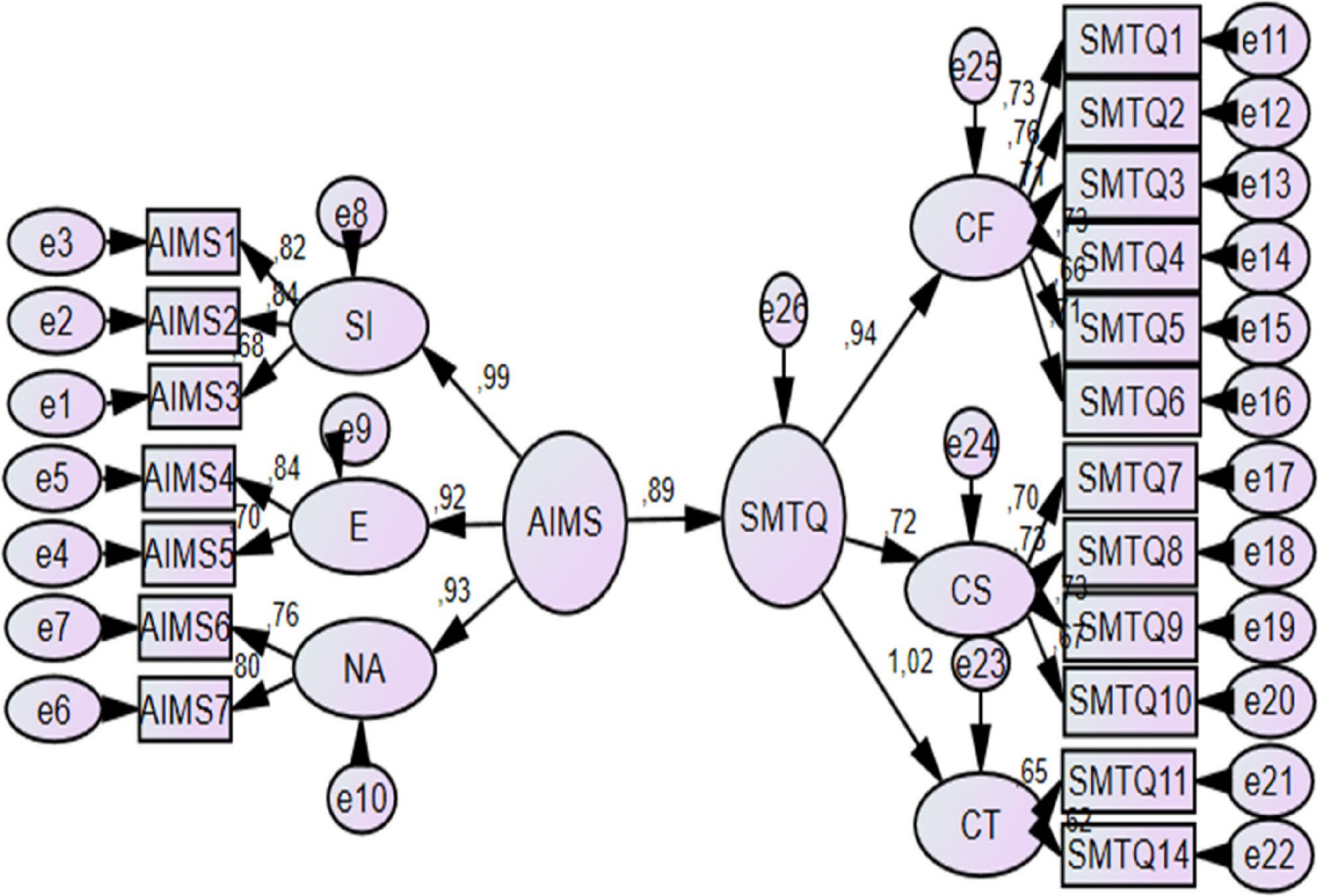
H1 test SEM analysis (AIMS→SMTQ)

Structural equation modeling was conducted to test H1, which proposed a direct relationship between athletic identity and mental toughness. The results of the SEM analysis are presented in Fig. 3; Table 5.

**Table 5:** Fit indices for H1 test SEM analysis (AIMS→SMTQ)

Compliance Indices	Reference Values			Value	Result	
	Good	Accept				
CMIN/DF	0 < χ^2^/SD ≤ 3	3 < χ^2^/SD ≤ 5		2.401	Good	
RMSEA	0 ≤ RMSEA≤0.05	0.05 ≤ RMSEA≤ 0.08		0.056	Acceptable	
GFI	0.90 < GFI ≤ 1	0.80 < GFI≤0.90		0.920	Good	
AGFI	0.90 < GFI ≤ 1	0.80 < GFI≤0.90		0.896	Acceptable	
CFI	0.95 < CFI ≤ 1	0.90 < CFI≤0.94		0.953	Good	
RMR	0 ≤ RMR≤0.05	0.05 ≤ SRMR≤0.10		0.040	Good	
TLI	0.95 < TLI ≤ 1	0.90 < TLI≤0.94		0.945	Good	
DF				145	—	
CMIN				348.084	—	

SEM Analysis Result						
Structural Relationship Status	Estimate(ß)	Standardize Estimate(ß)	S.E.	C.R.	*R* ^*2*^	*p*
SMTQ<---- AIMS	0.923	0.886	0.076	12.204	0.784	< .001

*Abbreviations*: *AIMS* Athletic Identity Measurement Scale, *SMTQ* Sport Mental Toughness Questionnaire, *SCI* Sport Confidence Inventory, *CFI* Comparative Fit Index, *TLI* Tucker–Lewis Index, *RMSEA* Root Mean Square Error of Approximation, *χ²/df* Chi-square divided by degrees of freedom, *α* Cronbach’s alpha

Fit indices indicated that the proposed model adequately represented the observed data (CMIN/DF = 2.401; RMSEA = 0.056; GFI = 0.920; AGFI = 0.896; CFI = 0.953; TLI = 0.945; RMR = 0.040). Fit indices were within recommended ranges. The standardized path coefficient from athletic identity to mental toughness was statistically significant (β = 0.886, *p* < .001), indicating a strong positive association between the two constructs. Based on these results, H1 was supported.

### Findings related to H2

**Fig. 4:**
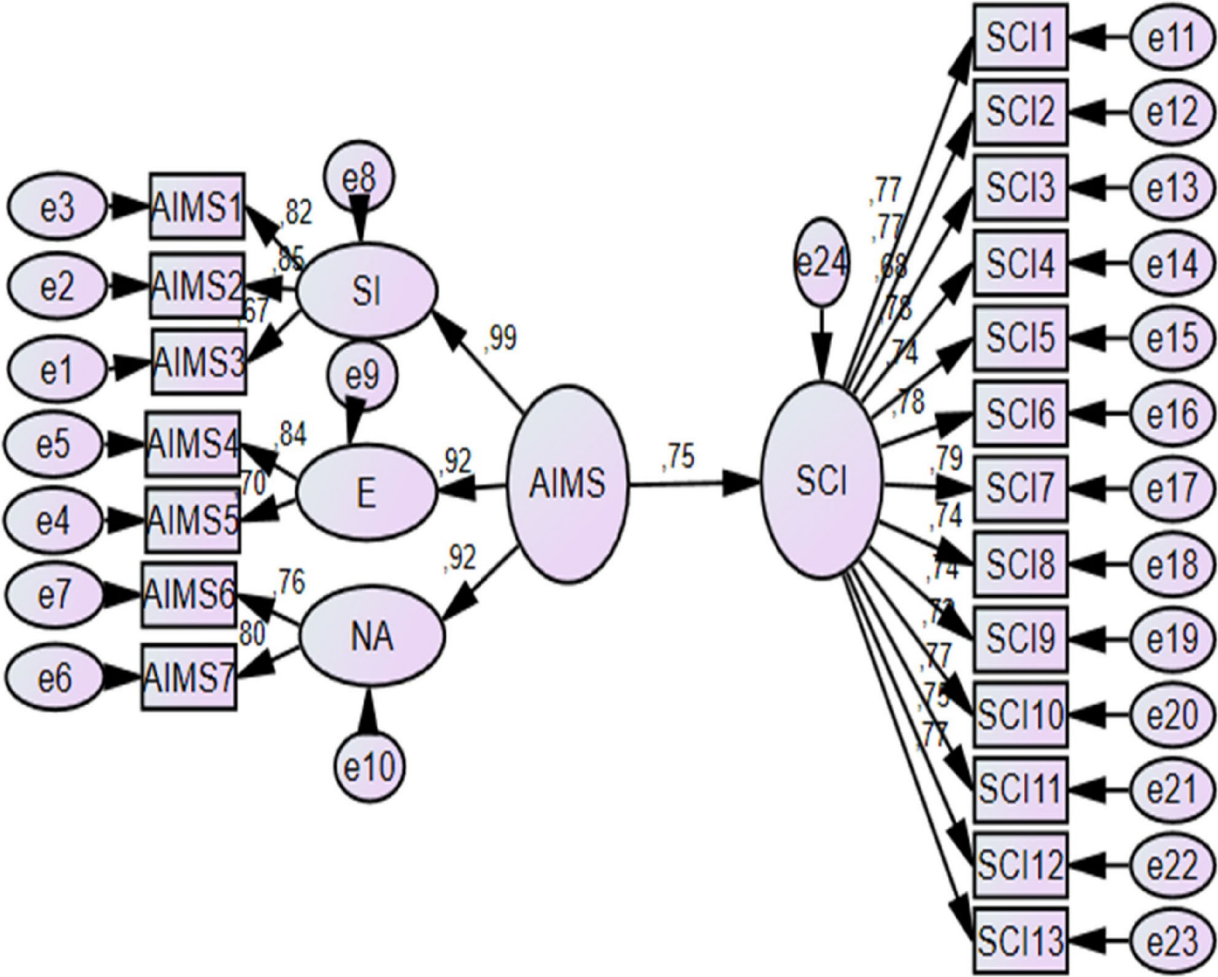
H2 test SEM analysis (AIMS → SCI)

Structural equation modeling was performed to test H2, which proposed a direct relationship between athletic identity and sport confidence. The results of the structural equation modeling analysis testing H2 are presented in Fig. 4 and Table 6.

**Table 6:** Fit indices for H2 Test SEM analysis (AIMS → SCI)

Compliance Indices	Reference Values			Value	Result	
	Good	Accept				
CMIN/DF	0 < χ^2^/SD ≤ 3	3 < χ^2^/SD ≤ 5		2.473	Good	
RMSEA	0 ≤ RMSEA≤0.05	0.05 ≤ RMSEA≤ 0.08		0.057	Acceptable	
GFI	0.90 < GFI ≤ 1	0.80 < GFI≤0.90		0.915	Good	
AGFI	0.90 < GFI ≤ 1	0.80 < GFI≤0.90		0.892	Acceptable	
CFI	0.95 < CFI ≤ 1	0.90 < CFI≤0.94		0.958	Good	
RMR	0 ≤ RMR≤0.05	0.05 ≤ SRMR≤0.10		0.032	Good	
TLI	0.95 < TLI ≤ 1	0.90 < TLI≤0.94		0.951	Good	
DF				166	—	
CMIN				410.557	—	

SEM Analysis Result						
Structural Relationship Status	Estimate(ß)	Standardize Estimate(ß)	S.E.	C.R.	*R* ^*2*^	*p*
SCI<---- AIMS	0.883	0.750	0.075	11.798	0.562	< .001

*Abbreviations*: *AIMS* Athletic Identity Measurement Scale, *SMTQ* Sport Mental Toughness Questionnaire, *SCI* Sport Confidence Inventory, *CFI* Comparative Fit Index, *TLI* Tucker–Lewis Index, *RMSEA *Root Mean Square Error of Approximation, *χ²/df* Chi-square divided by degrees of freedom, *α* Cronbach’s alpha

The model demonstrated acceptable fit to the data (CMIN/DF = 2.473; RMSEA = 0.057; GFI = 0.915; AGFI = 0.892; CFI = 0.958; TLI = 0.951; RMR = 0.032). All fit indices fell within recommended reference ranges, indicating that the proposed structural model adequately fit the data. The standardized path coefficient indicated a statistically significant positive association between athletic identity and sport confidence (β = 0.750, *p* < .001). Based on these findings, H2 was supported.

### Findings related to H3

**Fig. 5:**
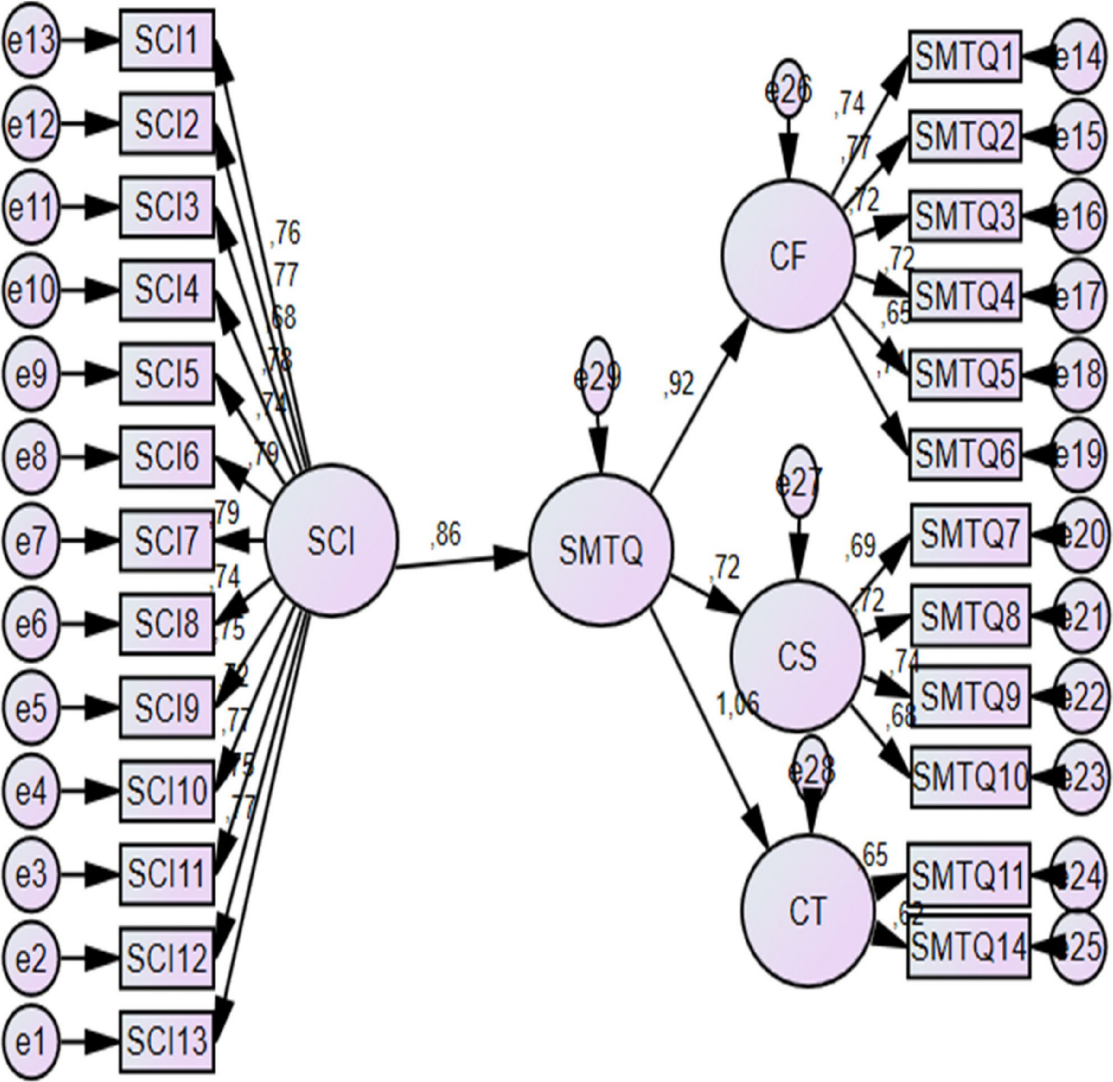
H3 test SEM analysis (SCI→SMTQ)

Structural equation modeling was conducted to test H3, which examined the direct relationship between sport confidence and mental toughness. The results of the SEM analysis are presented in Fig. 5; Table 7.

**Table 7:** Fit indices for H3 test SEM analysis (SCI → SMTQ)

Compliance Indices	Reference Values			Value	Result	
	Good	Accept				
CMIN/DF	0 < χ^2^/SD ≤ 3	3 < χ^2^/SD ≤ 5		2.292	Good	
RMSEA	0 ≤ RMSEA≤0.05	0.05 ≤ RMSEA≤ 0.08		0.054	Acceptable	
GFI	0.90 < GFI ≤ 1	0.80 < GFI≤0.90		0.898	Acceptable	
AGFI	0.90 < GFI ≤ 1	0.80 < GFI≤0.90		0.878	Acceptable	
CFI	0.95 < CFI ≤ 1	0.90 < CFI≤0.94		0.946	Good	
RMR	0 ≤ RMR≤0.05	0.05 ≤ SRMR≤0.10		0.036	Good	
TLI	0.95 < TLI ≤ 1	0.90 < TLI≤0.94		0.940	Acceptable	
DF				271	—	
CMIN				621.036	—	

SEM Analysis Result						
Structural Relationship Status	**Estimate(ß)**	**Standardize Estimate(ß)**	**S.E.**	**C.R.**	***R*** ^***2***^	***p***
SMTQ<---- SCI	0.766	0.861	0.056	13.630	0.741	< .001

*Abbreviations*: *AIMS* Athletic Identity Measurement Scale, *SMTQ* Sport Mental Toughness Questionnaire, *SCI* Sport Confidence Inventory, *CFI* Comparative Fit Index, *TLI* Tucker–Lewis Index, *RMSEA* Root Mean Square Error of Approximation, *χ²/df* chi-square divided by degrees of freedom, *α* Cronbach’s alpha

The model demonstrated acceptable fit to the data (CMIN/DF = 2.292; RMSEA = 0.054; GFI = 0.898; AGFI = 0.878; CFI = 0.946; TLI = 0.940; RMR = 0.036). Fit indices were within recommended ranges. The standardized path coefficient from sport confidence to mental toughness was statistically significant (β = 0.861, *p* < .001), indicating a strong positive association between the two variables. Based on these results, H3 was supported.

### Findings related to H4

**Fig. 6:**
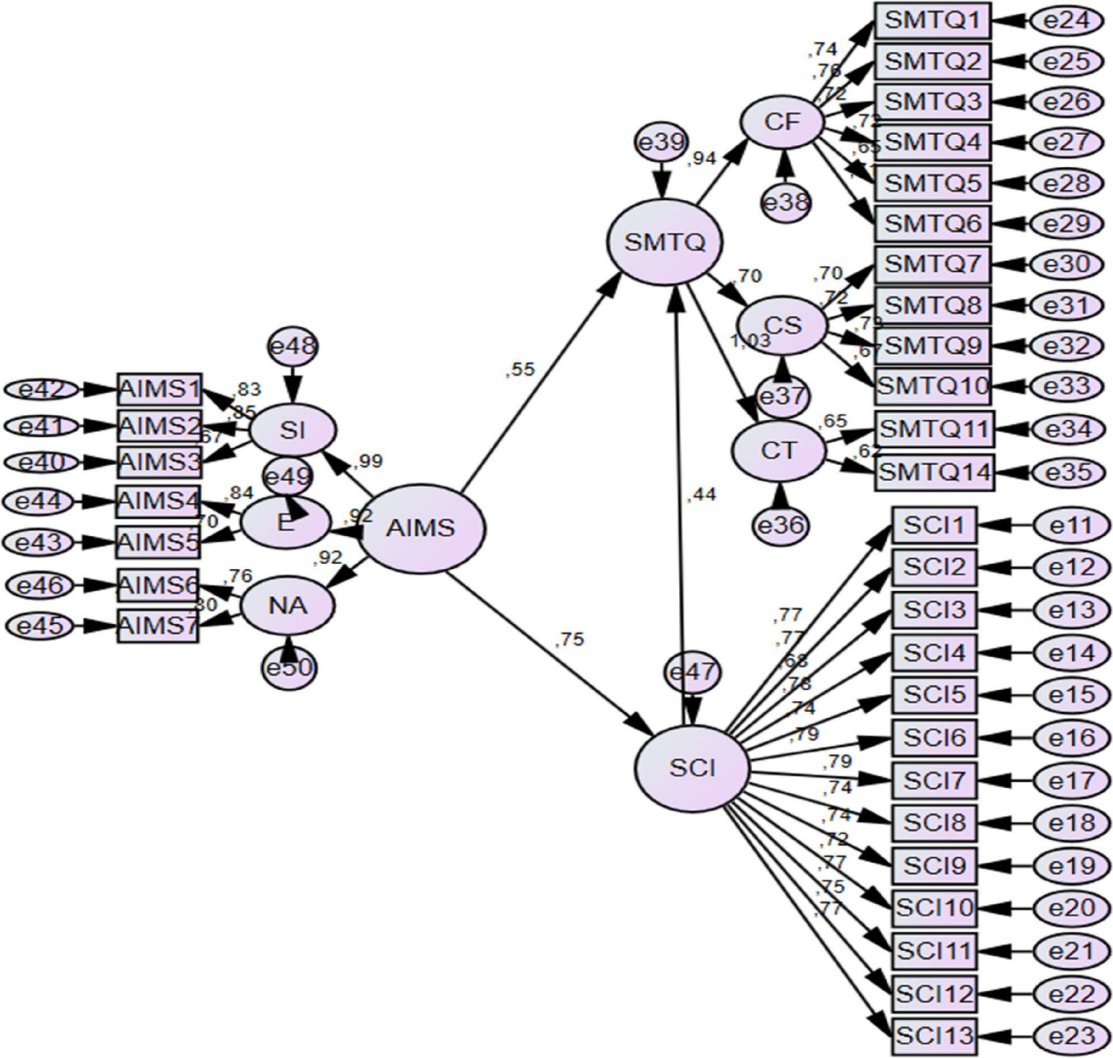
H4 test SEM analysis (AIMS→SCI→SMTQ)

Structural equation modeling was conducted to test H4, which proposed that sport confidence mediates the relationship between athletic identity and mental toughness. The results of the mediation model are presented in Fig. 6; Table 8.

**Table 8:** Fit indices for H4 test SEM analysis (AIMS → SCI → SMTQ)

Compliance Indices	Reference Values			Value	Result	
	Good	Accept				
CMIN/DF	0 < χ^2^/SD ≤ 3	3 < χ^2^/SD ≤ 5		2.181	Good	
RMSEA	0 ≤ RMSEA≤0.05	0.05 ≤ RMSEA≤ 0.08		0.051	Acceptable	
GFI	0.90 < GFI ≤ 1	0.80 < GFI≤0.90		0.876	Acceptable	
AGFI	0.90 < GFI ≤ 1	0.80 < GFI≤0.90		0.856	Acceptable	
CFI	0.95 < CFI ≤ 1	0.90 < CFI≤0.94		0.938	Good	
RMR	0 ≤ RMR≤0.05	0.05 ≤ SRMR≤0.10		0.037	Good	
TLI	0.95 < TLI ≤ 1	0.90 < TLI≤0.94		0.933	Good	
DF				455	—	
CMIN				992.463	—	

SEM Analysis Result						
Structural Relationship Status	Estimate(ß)	Standardized Estimate(ß)	S.E.	C.R.	*R* ^*2*^	*p*
SMTQ<---- AIMS	0.583	0.551	0.068	8.597	0.303	< .001
SMTQ<---- SCI	0.404	0.444	0.051	7.971	0.197	< .001
SCI<---- AIMS	0.875	0.751	0.073	11.944	0.765	< .001

*Abbreviations*: *AIMS* Athletic Identity Measurement Scale, *SMTQ* Sport Mental Toughness Questionnaire, *SCI* Sport Confidence Inventory, *CFI* Comparative Fit Index, *TLI* Tucker–Lewis Index, *RMSEA* Root Mean Square Error of Approximation, *χ²/df* Chi-square divided by degrees of freedom, *α* Cronbach’s alpha

The model showed satisfactory overall fit according to recommended goodness-of-fit criteria (CMIN/DF = 2.181; RMSEA = 0.051; GFI = 0.876; AGFI = 0.856; CFI = 0.938; TLI = 0.933; RMR = 0.037). All model fit indices were within recommended reference ranges, supporting the adequacy of the proposed mediation model.

The standardized path coefficients indicated that athletic identity significantly predicted sport confidence (β = 0.751, SE = 0.073, CR = 11.944, *p* < .001), and sport confidence significantly predicted mental toughness (β = 0.444, SE = 0.051, CR = 7.971, *p* < .001). In addition, the direct path from athletic identity to mental toughness remained statistically significant when sport confidence was included in the model (β = 0.551, SE = 0.068, CR = 8.597, *p* < .001). Compared to the direct effect observed in the initial model (β = 0.886; see Fig. 3; Table 5), the magnitude of the direct path from athletic identity to mental toughness was reduced in the mediation model but remained statistically significant. This pattern is statistically consistent with partial mediation; however, given the cross-sectional design, the result should be interpreted as associative rather than causal. Based on these results, H4 was supported. Alternative or competing structural models were not tested because the analysis followed a theory-driven model specification; future research may benefit from comparing alternative model structures.

## Discussion

### Identity dynamics

The findings of this study show that athletic identity is a strong predictor of mental toughness in female athletes. This result supports established theoretical perspectives positioning athletic identity as a core component of athletes’ motivational and self-regulatory systems [25]. When athletes internalize the athlete role, they develop patterns of competence, persistence, and emotional control that are fundamental to mental toughness.

Studies on early identity formation also stress that athletic identity is shaped by perceived competence, social reinforcement, and environmental expectations. Meta-analytic evidence further shows that athletes with stronger identity structures demonstrate greater intrinsic motivation and behavioral consistency [3]. The strong path coefficient in this study (β = 0.886) reinforces these findings, suggesting that athletic identity appears to function as a psychological anchor and is strongly associated with resilience-related characteristics.

In women’s sport, athletic identity may assume even greater importance due to gendered social norms and legitimacy pressures. Qualitative research indicates that self-esteem, perceived competence, and social validation shape how female athletes construct and protect athletic identity [18]. The present findings align with this literature by suggesting that athletes with stronger identity structures tend to display greater mental toughness, likely due to heightened emotional stability and more robust self-belief.

### Confidence mechanisms

Sport confidence emerged as a central mechanism explaining how athletic identity translates into resilience. Sport confidence reflects athletes’ expectations for successful performance and underpins emotional control, attentional focus, and coping efficiency [36]. Confident athletes typically exhibit stronger decision-making and more effective resource mobilization, qualities consistent with mental toughness. From an applied perspective, these findings indicate that psychological resources support adaptation to training load and performance-related stress. Athletic identity and sport confidence appear to function as internal regulatory mechanisms that enhance athletes’ capacity to tolerate cumulative physical and psychological demands. Athletes with stronger identity structures and higher confidence may be better equipped to maintain emotional stability, focus, and persistence during periods of intensified training or performance fluctuation. This adaptive capacity is particularly relevant in female athletes, for whom psychological stressors may interact with training demands and influence overall performance readiness.

The mediation results obtained in this study support the view that sport confidence influences mental toughness. Introducing sport confidence reduced—but did not eliminate—the direct effect of athletic identity on mental toughness (from β = 0.886 to β = 0.551), indicating partial mediation. This pattern corresponds with studies indicating that confidence forecasts persistence, emotional regulation, and stress tolerance [21]. Gender-specific research indicates that confidence distinctly influences the mental toughness profiles of female athletes [13]. These findings indicate that sport confidence may function as a cognitive pathway associated with mental toughness through which identity-driven beliefs become functional during stress. Given that female athletes often navigate competence-related stereotypes and evaluative pressures, confidence may play a potentially protective role in stabilizing mental toughness.

### Toughness and resilience in female athletes

Mental toughness is a multidimensional construct involving emotional regulation, persistence, and the ability to maintain focus under pressure. Studies indicate that female athletes frequently display unique toughness traits, such as enhanced emotional regulation and consistency, compared to their male counterparts [37]. Additional evidence suggests that structured sport participation enhances mental toughness by strengthening self-efficacy and stress management capacity [1].

This study enhances the existing literature by illustrating that athletic identity and sport confidence collectively influence mental toughness. This integrated psychological system—identity as the foundation, confidence as the driver, and toughness as the outcome—reflects models suggesting that resilience emerges through interactions between internal beliefs and external demands [10].

Importantly, the exclusive focus on female athletes strengthens the population-specific relevance of these findings. Psychological adaptation processes in women’s sport are shaped by unique sociocultural and organizational factors, including role legitimacy, visibility, and access to support systems. By modeling identity, confidence, and mental toughness together, this study contributes to a more nuanced understanding of resilience pathways in female athletic populations, aligning with calls for sex- and gender-sensitive approaches in sport science research.

### Female-specific psychological pressures

Female athletes face sociocultural pressures, including gender norms, emotional labor, and expectations from family and coaches, which shape their psychological functioning. These pressures can challenge identity clarity and elevate performance anxiety. Qualitative research indicates that women often manage the dual burden of maintaining performance identity while negotiating external expectations [18]. Research on mental health shows that psychological well-being affects female athletes’ performance, risk of injury, and recovery [14]. Biopsychosocial frameworks also stress that toughness comes from a mix of internal resources (like identity and confidence) and outside stressors [30]. The mediated pathway identified here reflects how female athletes organize psychological resources in complex sport environments.

### Clinical and practical relevance

In rehabilitation and return-to-play contexts, psychological readiness represents a critical yet often under-assessed component of recovery. While physical healing is essential, disruptions in athletic identity or reductions in sport confidence may compromise mental toughness, decision-making, and adherence to rehabilitation protocols. Female athletes, who may experience heightened evaluative pressures and identity-related stressors during injury, could be particularly affected by these psychological disruptions.

From a multidisciplinary perspective, the present findings highlight the importance of integrating psychological assessment into routine performance monitoring, rehabilitation planning, and athlete support systems [38]. Monitoring athletic identity and sport confidence alongside physical indicators may help practitioners identify athletes at risk for reduced mental toughness during high-stress periods such as injury rehabilitation, competitive deselection, or performance decline. Interventions aimed at reinforcing identity coherence and enhancing sport confidence may therefore complement physical training and rehabilitation strategies, supporting more holistic and gender-sensitive approaches in applied sport science and sports medicine settings.

From an applied perspective, the proposed model may also inform non-diagnostic psychological screening practices by identifying identity- and confidence-related vulnerability during periods of elevated physical and psychological stress. Interventions incorporating mental skills training, identity-supportive counseling, and confidence-enhancing strategies may contribute to safer return-to-play processes and improved long-term outcomes for female athletes.

For example, practitioners may use brief identity and sport confidence screening tools in routine athlete monitoring, or integrate structured confidence-building and identity-supportive exercises into mental skills training and rehabilitation programs.

### Limitations

This study has limitations. The cross-sectional design prevents causal inference. Self-report measures may reflect bias or subjective interpretations. Although the sample was large and diverse, generalizability may be limited to Turkish female athletes. Differences across sport types or competitive levels may influence psychological processes, suggesting the need for comparative and longitudinal research. Future studies incorporating longitudinal or intervention-based designs would strengthen causal interpretations and applied relevance. These design constraints are especially important when interpreting mediation and directional path estimates, which should be understood as statistical association patterns rather than evidence of causal or temporal ordering.

## Conclusion

This study provides evidence from an applied sport science perspective that athletic identity, sport confidence, and mental toughness form an interconnected psychological system in female athletes. Athletic identity strongly predicts mental toughness, and sport confidence partially mediates this association. These results emphasize the significance of gender-sensitive psychological frameworks and the necessity to facilitate identity development, bolster confidence, and fortify resilience in women’s sports. Future research should explore these relationships across different cultures, developmental stages, and performance levels to refine the model’s applicability.

## Data Availability

The data supporting the findings of this study are available from the corresponding author upon reasonable request. Due to ethical restrictions and the protection of participant confidentiality, individual-level raw data are not publicly available.

## Abbreviations

AI: Athletic identity
MT: Mental toughness
SC: Sport confidence
SEM: Structural equation modeling
CFA: Confirmatory factor analysis
AIMS: Athletic identity measurement scale
SMTQ: Sport mental toughness questionnaire
SCI: Sport confidence inventory
CFI: Comparative fit index
TLI: Tucker–lewis index
RMSEA: Root mean square error of approximation
